# Target Product Profile for a mobile app to read rapid diagnostic tests to strengthen infectious disease surveillance

**DOI:** 10.1371/journal.pone.0228311

**Published:** 2020-01-29

**Authors:** Rigveda Kadam, Wallace White, Nicholas Banks, Zachary Katz, Sabine Dittrich, Cassandra Kelly-Cirino

**Affiliations:** 1 Foundation for Innovative Diagnostics (FIND), Geneva, Switzerland; 2 Nuffield Department of Medicine, University of Oxford, Oxford, United Kingdom; McGill University Health Centre, CANADA

## Abstract

The essential role of rapid diagnostic tests (RDTs) in disease control is compromised every time a test is not performed correctly or its result is not reported accurately and promptly. A mobile app that utilizes the camera and connectivity of a common smartphone can fill this role of supporting the test’s proper execution and the automatic transmission of results. In a consensus process with 51 expert participants representing the needs of clinical users, healthcare programs, health information systems, surveillance systems, and global public health stakeholders, we developed a Target Product Profile describing the minimal and optimal characteristics of such an app. We collected feedback over two rounds and refined the characteristics to arrive at a preferred agreement level of greater than 75%, with an average of 92% agreement (range: 79–100%). As per this feedback, such an app should be compatible with many RDTs and mobile devices without needing accessories. The app should assist the user with RDT-specific instructions, include checks to facilitate quality control of the testing process and suggest results with ≥ 95% accuracy across common lighting conditions while allowing the user to determine the final result. Data from the app must be under the control of the health program that operates it, and the app should support at least one of the common data exchange formats HL7, FHIR, ASTM or JSON. The Target Product Profile also lays out the minimum data security and privacy requirements for the app.

## Introduction

Fit-for-purpose diagnostics are recognized by the World Health Organization (WHO) as having a key global role in plans ranging from eliminating malaria [1] to controlling antimicrobial resistance (AMR) [2,3]. A case in point is the malaria rapid diagnostic test (RDT), which, when used appropriately, has been shown to reduce the overuse of antimalarials [4,5]. However, insufficient training, lack of adherence to test instructions and inadequate quality assurance limit the impact of these widely used tests. As an example, in an external quality assessment of 1849 laboratory health workers reading malaria RDTs, interpretation errors with weak positives were 31.2% and with certain invalid tests were 32.5% [6]. Additionally, surveillance data from RDTs is crucial for local decision makers to evaluate malaria transmission and progress towards elimination, along with being critical in targeting AMR-related interventions in areas with high malaria-negative rates. For instance, the implementation of the malaria RDT has been linked, in cases with negative malaria results, to an increased use of antibiotics, shifting the risk of resistance from one class of drugs to another [7]. Decentralized data capture from RDTs for malaria as well as other infectious diseases such as dengue and influenza could enable countries to identify such emerging trends and implement corrective actions. However, most results from RDTs, when collected at all, are filed on paper, creating risks of delays, errors, and losses of data [8,9].

Some products to improve the accuracy of interpretation and the consistency of data capture are available now but not for the LMIC market. In high-income countries, companies offer RDT-reading instruments, which opto-electronically interpret lateral-flow tests and digitally communicate their results [10]. In low-resource settings, these readers are rare because of their costs and the additional requirement for health programs to manage supply chain logistics and ongoing maintenance for these devices, making them best suited to stationary deployments with high test volumes. An app that needs no hardware besides an ordinary smartphone, relying on its camera for photographic analysis of the test, can be adopted more broadly. Companies have recently commercialized a few such apps, some with FDA clearance to read colorimetric urinary tract infection assays [11,12] and others with CE mark to interpret quantitative lateral flow assays for fecal calprotectin [13–15]. Among apps that have passed review by a stringent regulatory authority, no app reads more than one type of RDT nor does any app read an infectious disease test. Mobile apps for malaria and other diseases of poverty have enabled operators to record results into health information systems (HIS) for surveillance, but none of these apps takes part in RDT interpretation [16–19].

To facilitate the development of tools that address some of the issues identified above, we developed a Target Product Profile (TPP) for a mobile phone app that aids an RDT user in interpreting and reporting the results. The app is intended to transmit RDT test data as well as patient data and contextual data such as the phone’s location to the relevant health program in order to strengthen surveillance in general and AMR surveillance in particular. The app is also expected to provide visibility into on-the-ground diagnostic practices.

## Methods

To develop this TPP, the previously established FIND and WHO process was followed [20–22]. A draft was developed (RK, WW) following a structured review of current practices and previously described needs. Each characteristic in the TPP has an optimal criterion that product developers should achieve if feasible and, in case the optimal is not feasible, a minimal criterion. To obtain consensus and arrive at a final TPP, we used a Delphi-like survey process. In order to include knowledge on state-of-the-art technology, participants also included product developers and stakeholders from adjacent industries of RDTs and HIS.

Participants rated each characteristic’s minimal and optimal criteria on a Likert scale (1 = strongly disagree, 2 = mostly disagree, 3 = neither agree nor disagree, 4 = mostly agree, 5 = strongly agree). We asked individuals to provide comments when they did not agree with a characteristic (Likert score of 1–3; in round 1, Likert score of 1–2), and they could opt to provide comments irrespective of the rating as well. Prior to conducting the survey, we specified consensus as > 50% of respondents agreeing with the proposed characteristics (Likert score of 4 or 5), with a second, preferred level of consensus at > 75% agreement. We analyzed the scores and comments in the first round of survey to inform revisions to the TPP, which we put out for a second survey round.

We planned to resolve any characteristics that did not achieve consensus through a meeting of the stakeholders most relevant to those characteristics.

## Results

### Content of the TPP

The completed TPP contains 28 characteristics organized into seven sections (S1 Document).

#### Scope of the app

This app is intended for use by a broad set of front-line health workers who use RDTs, and it should be easy for them to learn and use. Suggesting a result rather than determining it was chosen to make the app helpful to its users without changing the users’ responsibility.

#### System components

The app should be compatible with as many phones and RDTs as possible, optimally without needing any additional components like a stand.

#### Functional requirements

In addition to having relevant language and workflow characteristics, the app should assist the user by giving instructions on use of the RDT and checking quality controls of the RDT and camera. The app should automatically report, to the degree the health program chooses, data to enable case management, epidemiology, and supervision.

#### Operational requirements

The app should work in the varying lighting conditions where users are running RDTs.

#### Data characteristics

Recognizing that the app handles highly sensitive data that are not owned by the app company, the app must be configurable so that health programs can operate it in compliance with applicable policies and regulations, and the app must interface with the health program’s chosen servers over common protocols. Offline use must be supported.

#### Performance requirements

For the result suggestion feature to be useful, it must be highly accurate across all types of results.

#### Pricing and accessibility

The app has to be highly accessible to each health program to be adopted.

### Participant demographics

Of the 110 people invited, the first round and second round received responses from 44 (40%) and 28 (25%), respectively, with 21 (19%) completing both rounds. The 51 total participants (46%) represented a variety of organizations and stakeholders with interests in global health diagnostics and surveillance, and experience in all WHO regions (Tables A and B in S2 Document). In each of the four fields of expertise surveyed, at least 30% of the group reported five or more years of experience, with HIS having the highest percentage of such respondents (53%) (Table C in S2 Document).

### Delphi survey

In Round 1, the draft TPP (S3 Document) was met with basic consensus (> 50% agreement) for all 26 characteristics, but six minimal and 11 optimal criteria did not meet the preferred consensus level (> 75%), as shown in Fig A in S2 Document. We revised all of the characteristics that did not achieve preferred consensus and six additional characteristics, in total revising 18 of 26 characteristics, and we added one, based on common themes in the feedback.

**Fig 1:**
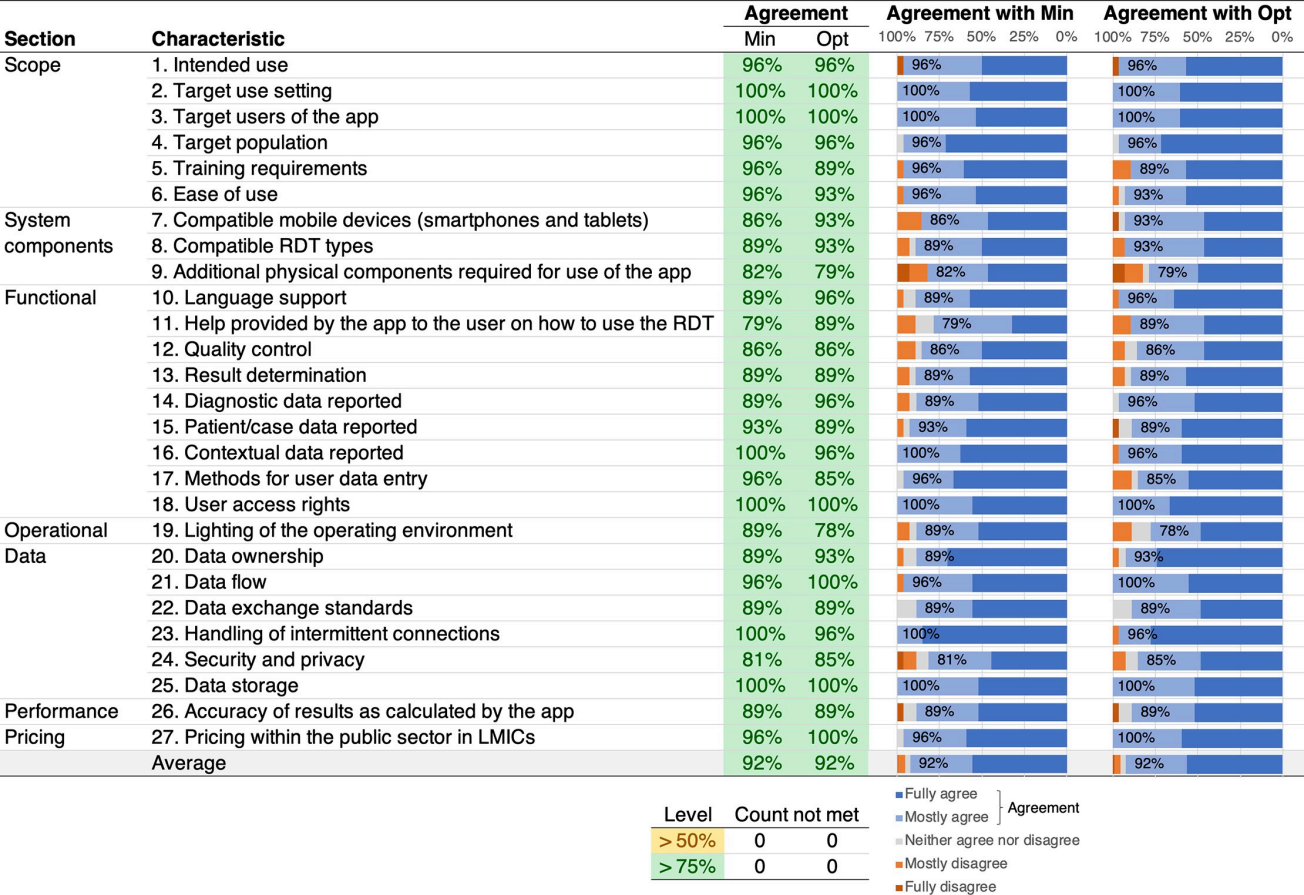
Agreement levels and distributions for the minimal ("Min") and optimal ("Opt") criteria of each TPP characteristic in round 2.

In Round 2, this revised TPP (S4 Document) achieved preferred consensus for both minimal and optimal of all characteristics, as shown in Fig 1.

Given the high level of consensus, a TPP consensus meeting as described in the Methods was not required, but it was deemed necessary to make three changes to address issues raised in the second round. The first two changes are minor and were not reviewed further:

- **Language support.** Participants noted the importance of additional languages being part of the minimal criterion and the relatively low burden for an app developer to provide them. We increased the minimal languages to six to represent more of the variety used in LMICs, including languages with diverse characteristics that need to be considered in the app’s design.
- **RDT workflow support.** In response to survey participants’ suggestions on having the app support running several RDTs at a time in a batch, we created this new characteristic. The optimal level requires support for batch and individual workflows, while the minimal can be met by an app limited to reading one RDT at a time.

The third change was more complex:

c **Security and privacy.** Among the participants who participated in both rounds, this was the only characteristic whose optimal agreement was lower in the second round, and its minimal agreement fell in the second round more than any other characteristic (Fig B in S2 Document). Participants pointed out that the EU’s General Data Protection Regulation (GDPR) [23], while containing elements that can be useful for this product, cannot be applied as a whole outside the EU and, in fact, has elements which would conflict with the laws and policies of some LMICs. We consulted with each of the 10 participants who had commented in either round on GDPR, leading to a revision that adapts the six principles of GDPR to this product without requiring compliance with GDPR in its entirety. Put to a vote, this new text was approved by a vote of eight for and none against (two did not respond).

Additional feedback relating to certain characteristics has been summarized in Table D in S1 Document with the intent of providing useful information to product developers.

## Discussion

This TPP incorporates perspectives of diverse organizations involved in policy, funding, advocacy, provision of care, research, and product development. The employed methodology enabled us to include nuanced feedback across all the major characteristics, especially around difficult topics such as data security and privacy. As a result, this TPP is a first-of-its-kind guide articulating the product requirements for RDT-reading apps for LMICs. It describes a range of potential products represented between its minimal characteristics, which are deemed acceptable and technologically feasible, and its optimal characteristics, which are more desirable but more challenging to deliver. We intend for this document to serve as an informative tool for a variety of stakeholders, such as app developers, donors and policymakers.

For the benefit of product developers and other stakeholders, we have also listed significant disagreements that arose during development of this TPP:

### Intended use and accuracy

To yield as much data as possible for surveillance, one approach is to set the app’s capabilities so that it will not need a regulatory authorization as an in vitro diagnostic device. The current regulatory frameworks would likely limit an app that definitively determined results to the specific RDTs and phones used in its regulatory studies, precluding use with the hundreds of other RDTs and mobile phones in use. Some people would accept that restriction provided that such an app can improve RDT interpretation accuracy, but whether apps are more accurate than the eye has not been established. Some survey participants cautioned that interpretation of faint positives is at least as difficult for an app as for a well-trained person, particularly under this app’s diverse lighting conditions. By eliminating simple errors like overlooking a missing control line, however, an app may perform as well or better than real-world users with limited training and other demands on their attention. Such an app has not previously undergone a regulatory review. Therefore, product developers should aim to give health programs the ability to disable the result suggestion feature to adapt the app to in-country regulations.

### Burden versus benefits

Improved surveillance depends on sustained use of the app as a reporting tool. The TPP’s brief characteristic on ease of use, which belies the complexity of that topic given the lack of baseline metrics from disparate settings, is but one part of this broader consideration. The app must offer enough benefits to justify its use. Current practice in some settings does not require reporting of negative results; no matter how user-friendly, no app can be less work than that. The more text and photos the user must submit to help the data recipients, the greater the challenge of getting busy healthcare workers to open the app for every test. The app should therefore be configurable by each health program to collect only the fields that program needs. Beyond minimizing burden, helping users care for their patients by interpreting tests accurately is a logical way to encourage use. When a result does have to be reported, the app can save time compared to paper by automatically collecting contextual data. The app’s data will also enable health programs to monitor the frequency of use and provide incentives for consistent use.

### The Bring Your Own Device (BYOD) model

The issue of supporting mobile devices owned by users, or the related issue of supporting devices already deployed by health programs for other purposes, needs consideration. Supporting almost any device should promote the app’s adoption, but the diversity of devices in these settings will be a challenge to ensuring good performance. A physical component like an optical calibration card, by enabling performance checks and corrections for each phone, might solve that problem but create another: that of supplying and maintaining the cards in good condition. Ideally, the app would check each phone’s performance without needing any such accessories. Survey participants also expressed concern that too few users would have sufficiently capable devices for BYOD to be viable.

### Personal data management

The handling of personal data concerning health requires special care and will need to be factored into the app’s development. One alternative is not to include patient names, patient identifiers, or any other personal data in the app. This would still enable surveillance but leave the problem of reporting results for patient records elsewhere. A second option is to structure this product to interoperate with other apps that take care of any personal data. Such a product, in the form of an app or a software library, would take care of the RDT reading steps, while another app would collect patient data and report results to a server, as some apps already do.

### Diverse data policies

While stakeholders agree that this app must respect local approaches to data ownership, security, and storage, it is not simple to summarize those needs. Many jurisdictions have not published policies on them. Whether published or not, practices diverge considerably. Some countries will require that all data stay within their borders, for instance, while others will prefer to rely on the cloud. The app must be configurable to support the practices of each setting.

### Pricing

As a nascent product category, an RDT-reading app does not have the established market and economic models of a malaria RDT or a tuberculosis test. Some participants called for the app to be free to use in these settings, as are some digital health software products and services. While donor funding may partially support the development of this app, the ongoing tasks of maintaining the app and supporting its users will have to be covered. An inexpensive pricing model, as suggested by other participants, would provide for this and could be part of a healthy market that encourages other app developers to offer innovations. Future work is needed to develop an approach that delivers value and is sustainable.

The primary limitation of the described work is a particular aspect of the survey approach. By requiring comments whenever a participant did not agree, which helped us understand the reasons for not agreeing, the survey could have encouraged agreement as the quickest route to completion. Consistent with that idea, when we made comments obligatory in round 2 for responses of “neither agree nor disagree,” the fraction of “neither” responses did decrease overall (compare Fig A in S2 Document and Fig 1), and agreement levels increased slightly among characteristics that had not changed (Fig B in S2 Document). Participant comments, a valuable output of the surveys, should be encouraged. Given the many optional comments submitted regardless of Likert score, however, it is worth considering whether it is appropriate to require comments only in cases of non-agreement.

We hope this TPP will promote the development of effective, useful, sustainable apps to support healthcare workers in interpreting and recording RDT results and to support healthcare programs in improved surveillance, particularly with a view on AMR-related data paucity.

## Supporting information

S1 Document Final TPP. (PDF)

S2 Document Survey tables and figures. (PDF)

S3 Document Draft TPP round 1. As used in feedback round 1.
(PDF)

S4 Document Draft TPP round 2. As used in feedback round 2.
(PDF)

## References

[1] World Health Organization. Malaria Threats Map [Internet]. [cited 6 Aug 2019]. Available: https://apps.who.int/malaria/maps/threats/

[2] World Health Organization. Global action plan on antimicrobial resistance [Internet]. 2015. Available: http://www.who.int/antimicrobial-resistance/publications/global-action-plan/en/10.7196/samj.9644 26242647

[3] World Health Organization. No Time to Wait: Securing the future from drug-resistant infections [Internet]. 2019 Apr. Available: http://www.who.int/antimicrobial-resistance/interagency-coordination-group/final-report/en/

[4] Odaga J, Sinclair D, Lokong JA, Donegan S, Hopkins H, Garner P. Rapid diagnostic tests versus clinical diagnosis for managing people with fever in malaria endemic settings. Cochrane Database Syst Rev. 2014; 10.1002/14651858.CD008998.pub2 24740584 PMC4468923

[5] D’Acremont V, Kahama-Maro J, Swai N, Mtasiwa D, Genton B, Lengeler C. Reduction of anti-malarial consumption after rapid diagnostic tests implementation in Dar es Salaam: a before-after and cluster randomized controlled study. Malar J. 2011;10: 107 10.1186/1475-2875-10-107 21529365 PMC3108934

[6] Mukadi P, Gillet P, Lukuka A, Mbatshi J, Otshudiema J, Muyembe J-J, et al External Quality Assessment of Reading and Interpretation of Malaria Rapid Diagnostic Tests among 1849 End-Users in the Democratic Republic of the Congo through Short Message Service (SMS). PLOS ONE. 2013;8: e71442 10.1371/journal.pone.0071442 23967211 PMC3742745

[7] Hopkins H, Bruxvoort KJ, Cairns ME, Chandler CIR, Leurent B, Ansah EK, et al Impact of introduction of rapid diagnostic tests for malaria on antibiotic prescribing: analysis of observational and randomised studies in public and private healthcare settings. BMJ. 2017;356: j1054 10.1136/bmj.j1054 28356302 PMC5370398

[8] Cori Anne, Donnelly Christl A., Dorigatti Ilaria, Ferguson Neil M., Fraser Christophe, Garske Tini, et al Key data for outbreak evaluation: building on the Ebola experience. Philos Trans R Soc B Biol Sci. 2017;372: 20160371 10.1098/rstb.2016.0371 28396480 PMC5394647

[9] Kanal K, Chou TL, Sovann L, Morikawa Y, Mukoyama Y, Kakimoto K. Evaluation of the proficiency of trained non-laboratory health staffs and laboratory technicians using a rapid and simple HIV antibody test. AIDS Res Ther. 2005;2: 5 10.1186/1742-6405-2-5 15907202 PMC1156864

[10] Faulstich K, Gruler R, Eberhard M, Lentzsch D, Haberstroh K. Handheld and Portable Reader Devices for Lateral Flow Immunoassays In: Wong R, Tse H, editors. Lateral Flow Immunoassay. Totowa, NJ: Humana Press; 2009 pp. 1–27. 10.1007/978-1-59745-240-3_9

[11] 510(k) premarket notification K170118: Scanostics UTI Check Application Test System [Internet]. U.S. Food and Drug Administration; 2017 Sep. Available: https://www.accessdata.fda.gov/scripts/cdrh/cfdocs/cfpmn/pmn.cfm?ID=K170118

[12] 510(k) premarket notification K173327: DIP/U.S. Urine Analysis Test System [Internet]. U.S. Food and Drug Administration; 2018 Jul. Available: https://www.accessdata.fda.gov/scripts/cdrh/cfdocs/cfpmn/pmn.cfm?ID=K173327

[13] Heida A, Knol M, Kobold AM, Bootsman J, Dijkstra G, van Rheenen PF. Agreement Between Home-Based Measurement of Stool Calprotectin and ELISA Results for Monitoring Inflammatory Bowel Disease Activity. Clin Gastroenterol Hepatol. 2017;15: 1742–1749.e2. 10.1016/j.cgh.2017.06.007 28606846

[14] Vinding KK, Elsberg H, Thorkilgaard T, Belard E, Pedersen N, Elkjaer M, et al Fecal Calprotectin Measured By Patients at Home Using Smartphones—A New Clinical Tool in Monitoring Patients with Inflammatory Bowel Disease. Inflamm Bowel Dis. 2016;22: 336–344. 10.1097/MIB.0000000000000619 26535869

[15] Haisma S-M, Galaurchi A, Almahwzi S, Balogun JAA, Kobold ACM, Rheenen PF van. Head-to-head comparison of three stool calprotectin tests for home use. PLOS ONE. 2019;14: e0214751 10.1371/journal.pone.0214751 30998692 PMC6472756

[16] Population Services International (PSI). Malaria Case Surveillance Application [Internet]. [cited 20 May 2019]. Available: https://mis.psi.org/malaria-case-surveillance-application/?lang=en

[17] Meyers DJ, Ozonoff A, Baruwal A, Pande S, Harsha A, Sharma R, et al Combining Healthcare-Based and Participatory Approaches to Surveillance: Trends in Diarrheal and Respiratory Conditions Collected by a Mobile Phone System by Community Health Workers in Rural Nepal. PLoS ONE. 2016;11 10.1371/journal.pone.0152738 27111734 PMC4844116

[18] Francis F, Ishengoma DS, Mmbando BP, Rutta ASM, Malecela MN, Mayala B, et al Deployment and use of mobile phone technology for real-time reporting of fever cases and malaria treatment failure in areas of declining malaria transmission in Muheza district north-eastern Tanzania. Malar J. 2017;16: 308 10.1186/s12936-017-1956-z 28764792 PMC5540449

[19] Olson D, Lamb M, Lopez MR, Colborn K, Paniagua-Avila A, Zacarias A, et al Performance of a Mobile Phone App-Based Participatory Syndromic Surveillance System for Acute Febrile Illness and Acute Gastroenteritis in Rural Guatemala. J Med Internet Res. 2017;19 10.2196/jmir.8041 29122738 PMC5701088

[20] Denkinger CM, Dolinger D, Schito M, Wells W, Cobelens F, Pai M, et al Target Product Profile of a Molecular Drug-Susceptibility Test for Use in Microscopy Centers. J Infect Dis. 2015;211: S39–S49. 10.1093/infdis/jiu682 25765105 PMC4425821

[21] Dittrich S, Tadesse BT, Moussy F, Chua A, Zorzet A, Tängdén T, et al Target Product Profile for a Diagnostic Assay to Differentiate between Bacterial and Non-Bacterial Infections and Reduce Antimicrobial Overuse in Resource-Limited Settings: An Expert Consensus. PLOS ONE. 2016;11: e0161721 10.1371/journal.pone.0161721 27559728 PMC4999186

[22] A Multiplex Multi-Analyte Diagnostic Platform [Internet]. FIND, MSF, and WHO; 2018 Mar. Available: https://www.who.int/medical_devices/TPP_20180327_final.pdf

[23] Regulation (EU) 2016/679 of the European Parliament and of the Council of 27 April 2016 on the protection of natural persons with regard to the processing of personal data and on the free movement of such data, and repealing Directive 95/46/EC (General Data Protection Regulation) [Internet]. Official Journal of the European Union 5 4, 2016 Available: https://eur-lex.europa.eu/eli/reg/2016/679/oj

