# Supplementary material for: Target Product Profile for a mobile app to read rapid diagnostic tests to strengthen infectious disease surveillance
Source: PLoS One. 2020 Jan 29;15(1):e0228311. doi: 10.1371/journal.pone.0228311 (PMC6988927; doi:10.1371/journal.pone.0228311)
Supplement: S1 Document — (PDF) [file pone.0228311.s001.pdf]

Supporting Information 1: Final TPP

Characteristic	Minimal	Optimal	Notes
**Scope of the app**			
1. Intended use	The app, after photographing each RDT, will suggest an interpretation of the test to the user, who can either accept that or choose an alternate interpretation. The app will transmit these test data as well as patient data entered by the user and contextual data such as the phone's location to the health program.		
2. Target use setting	Community outreach (Level 0) and primary care (Level 1)[1]		
3. Target users of the app	Community health workers with minimal training and any health worker with a similar or superior training level		
4. Target population	Total population presenting for care at relevant settings		
5. Training requirements	Less than 2 hours of in-person training	Same plus an option for users to train themselves (without a teacher). This could be done via the app itself or by providing web-based training resources	Assuming the users already use RDTs and mobile devices
6. Ease of use	Not substantially more burdensome than using paper forms for equivalent record-keeping	Less burdensome than using paper forms for equivalent record-keeping	

[1] Ghani AC, Burgess DH, Reynolds A, Rousseau C. Expanding the role of diagnostic and prognostic tools for infectious diseases in resource-poor settings. Nature 2015;528:S50-52

Characteristic	Minimal	Optimal	Notes
**System components**			
7. Compatible mobile devices (smartphones and tablets)	The app maker shall publish and maintain a list of ≥ 10 models of Android mobile devices that are readily available in LMICs	Same plus<br><br>Most Android mobile devices with a rear-facing camera and flash that are readily available in LMICs<br><br>The app maker shall publish a way (such as an optical calibration functionality) for users to confirm the compatibility of any device.<br><br>Devices using the app shall remain functional for other apps and uses.	The minimal is intended to support scenarios in which LMIC health systems provide mobile devices to their health care workers.<br><br>The optimal is intended to support less-controlled scenarios, including “bring your own device” (BYOD). The optimal app shall not require a “dedicated device” or “lock task mode” as defined in Android.[2]
8. Compatible RDT types	Qualitative lateral flow tests<br><br>The app shall be compatible with conventional RDTs of many brands and types, not requiring special versions or packages.	Same plus<br><br>Semi-quantitative (threshold) lateral flow tests	These semi-quantitative tests involve comparison of the intensity of a test line to a reference
9. Additional physical components required for use of the app	Acceptable if they are highly portable and nearly universal (not specific to a small number of mobile devices or RDTs)	None	Examples: a stand or an optical calibration target. Dependence on these is not optimal since they would have to be supplied and maintained for extended periods.

[2] Dedicated Devices Overview, Android Developers Documentation. https://developer.android.com/work/dpc/dedicated-devices/ retrieved 29 January 2019.

Characteristic	Minimal	Optimal	Notes
**Functional requirements**			
10. Language support	Arabic, English, French, Portuguese, Spanish, and Swahili	Same plus<br><br>The app can be configured by the health program to include a local language or languages	
11. RDT workflow support	Serial (one RDT at a time)	Serial and parallel (several RDTs at a time in a batch)	
12. Help provided by the app to the user on how to use the RDT	The app shall include a countdown timer to prompt the user to read the RDT after development. The user shall be responsible for setting the duration of this timer appropriately.	The app will provide the user with access to RDT instructions equivalent to Quick Reference Instructions (QRI) or a job aid.<br><br>The app shall include a countdown timer to prompt the user to read the RDT(s) after development. The app shall set the duration according to each RDT's QRI or job aid.	Minimal: Users should follow regular instructions for operation of the RDT, just like they should without this app.<br><br>Regardless of this characteristic, the app will provide instructions for use of the app, such as how to photograph the RDT and how to use gloves to avoid contamination of the mobile device.
13. Quality control	• Check of elapsed time to reading of result<br>• Check of the RDT's internal control(s)<br>• Check of sufficient capability of the mobile device's camera	Same plus<br><br>• Analysis of background to check sufficient washing of the sample by the buffer<br>• Check of expiration by date	Failures of these checks will result in warnings to the user and in the record

Characteristic	Minimal	Optimal	Notes
14. Result determination	The app suggests the result and requires the user to confirm or override. If the user overrides, the app asks the user to provide a reason.		The app should make it easy to provide a reason that will be useful for subsequent review. For example, the app could allow selection from a short list or entry in an "other reason" text field.
15. Diagnostic data reported	• Brand and type of test (possibly by photographing the test and its packaging)<br>• Underlying values and outcome of quality controls (e.g. elapsed time value and whether the time was in the expected range or not)<br>• Result as selected by user<br>• Result as calculated by the app<br>• Photograph used to calculate result, without patient-identifiable information, to be transmitted for select cases as determined by the health program (e.g. discordance between results determined by the app and the user)	Same plus<br><br>• Lot number and expiration date of the test (possibly by photographing the test and its packaging)<br>• RDT instruction version<br>• Intermediate data and parameters used to calculate the result (e.g. intensities of test line and control line)<br>• Other relevant diagnostic data as inputted by the user	By removing patient-identifiable information, which might be written on the RDT, from the photograph of the RDT, the photograph can more easily be managed and used for non-clinical purposes like quality control. For example, the app might crop the photo to show only the test strip.
16. Patient/case data reported	• As determined by the health program (e.g. patient identification, patient location, patient consent)		
17. Contextual data reported	• User identification<br>• Location of test (if enabled by the health program)<br>• Time and date of test<br>• Model of mobile device<br>• OS version<br>• App version		

Characteristic	Minimal	Optimal	Notes
18. Methods for user data entry	• Typing<br>• Scanning 1D and 2D barcodes		The user can choose from these methods when entering the data types listed above
19. User access rights	Provides access to specific data and app features for users with different roles		Roles may include data manager (facility supervisor) or RDT user (health care worker)
**Operational requirements**			
20. Lighting of the operating environment	Any setting in which the user can see well enough to run the test.<br><br>Infrequently, the app may tell the user that it cannot operate in the current lighting. If the user continues using the app without moving to a better-lit location, the app will not suggest a result; it will capture and transmit only the user's interpretation of the result and a photograph of the RDT.		Example settings:<br>• dim indoors without artificial lighting<br>• window-less indoors with fluorescent lighting<br>• mixed lighting<br>• outdoors in direct sun<br>• outdoors in dappled, moving shadows from a tree<br>• outdoors in shade with indirect sunlight off a red wall
**Data characteristics**			
21. Data ownership	Data ownership shall be in compliance with the in-country regulations		
22. Data flow	De-identified output data can be exchanged with different authorities with authorization by local authorities		
23. Data exchange standards	The app supports at least one of the following formats: HL7, FHIR, ASTM or JSON	The app supports all of the following formats: HL7, FHIR, ASTM and JSON	For connections to systems such as LISs, DHIS2, EHRs, national registries and surveillance systems
24. Handling of intermittent connections	The user shall be able to perform tests offline, in which case the app shall transmit that data when back online	Same plus<br><br>The app shall synchronize automatically (without user action) in the background when back online	

Characteristic	Minimal	Optimal	Notes
25. Security and privacy	To facilitate use by health programs in accordance with the laws, regulations and policies of their settings and following best practices, the app shall provide configurable features so that personal data can be<br>(a) gathered transparently to users and patients, including consent<br>(b) collected and processed only for purposes compatible with the health program's purposes<br>(c) limited to what is relevant and necessary<br>(d) collected accurately<br>(e) stored in identifiable form no longer than necessary<br>(f) secured for integrity and confidentiality, with encryption at rest and in transmission		(a)–(f) have been adapted from the EU General Data Protection Regulation 2016/679 (GDPR), article 5, sec. 1. Note that not all of the GDPR is relevant or appropriate to this product in these settings.
26. Data storage	The health program shall be able to choose the destination of the app's data		
**Performance requirements**			
27. Accuracy of results as calculated by the app (for non-clinical purposes)	≥ 95% concordance with an expert user	≥ 98% concordance with an expert user	
**Pricing and accessibility**			
28. Pricing within the public sector in LMICs	The pricing structure should be adapted to LMICs (including open-source and open-access solutions), and strategies for further cost reductions should be in place		Details to be established in a Global Access agreement. For applicable markets, see https://www.finddx.org/find-negotiated-product-pricing/
