# Supplementary material for: Target Product Profile for a mobile app to read rapid diagnostic tests to strengthen infectious disease surveillance
Source: PLoS One. 2020 Jan 29;15(1):e0228311. doi: 10.1371/journal.pone.0228311 (PMC6988927; doi:10.1371/journal.pone.0228311)
Supplement: S2 Document — (PDF) [file pone.0228311.s002.pdf]

Supporting Information 2: Survey tables and figures

Table A: Count of respondents by their role in this field

Type of respondent	Count	Percent
International agency or health program	12	24%
Industry: HIS	12	24%
Academic: other than RDT apps	10	20%
Industry: RDT apps	6	12%
Global health consultant	5	10%
Industry: RDTs	4	8%
Academic: researching RDT apps	2	4%
Total	51	

Table B: Respondents' regions of experience, according to regions defined by the World Health Organization [39]

Region	Count	Percent
African Region	46	90%
South-East Asia Region	37	73%
Region of the Americas	24	47%
Western Pacific Region	13	25%
European Region	14	27%
Eastern Mediterranean Region	9	18%

Table C: Respondents' fields of experience

	Years of experience (Count and Percent)									
Field	None		< 1 year		1–5 years		5–10 years		> 10 years	
Clinical practice	30	59%	0	0%	5	10%	7	14%	9	18%
Clinical research	18	35%	3	6%	7	14%	10	20%	13	25%
Diagnostic product development	17	33%	0	0%	14	27%	8	16%	12	24%
HIS	10	20%	3	6%	11	22%	14	27%	13	25%

Fig A: Agreement levels for each TPP characteristic in Round 1

Section	Characteristic	Agreement Min	Agreement Opt	Agreement with Min	Agreement with Opt
Scope	1. Intended use	91%	70%	91%	70%
	2. Target use setting	98%	86%	98%	86%
	3. Target users of the app	98%	89%	98%	89%
	4. Target population	91%	86%	91%	86%
	5. Training requirements	75%	73%	75%	73%
	6. Ease of use	86%	86%	86%	86%
	7. Required system components for the app to be used	73%	70%	73%	70%
	8. Compatible mobile devices	84%	77%	84%	77%
	9. Compatible RDT types	91%	75%	91%	75%
Functional	10. RDT instructions given by the app	57%	80%	57%	80%
	11. Quality control	84%	77%	84%	77%
	12. Result determination	86%	75%	86%	75%
	13. Diagnostic data reported	80%	82%	80%	82%
	14. Patient/case data reported	77%	68%	77%	68%
	15. Contextual data reported	93%	89%	93%	89%
	16. Methods for user data entry	93%	77%	93%	77%
	17. User access rights	82%	84%	82%	84%
Operational	18. Lighting of the operating environment	86%	84%	86%	84%
Data	19. Data ownership	64%	59%	64%	59%
	20. Data flow	84%	86%	84%	86%
	21. System access (public API)	77%	68%	77%	68%
	22. Handling of intermittent connections	98%	95%	98%	95%
	23. Security and privacy	84%	84%	84%	84%
	24. Data storage	73%	55%	73%	55%
Performance	25. Accuracy of results as calculated by the app	70%	68%	70%	68%
Pricing	26. Pricing within the public sector in LMICs	77%	75%	77%	75%
	Average	83%	78%	83%	78%

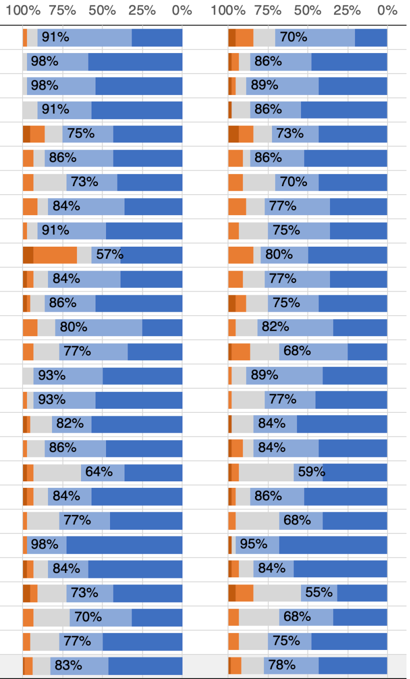

Level	Count not met	
> 50%	0	0
> 75%	6	11

Fully agree
Mostly agree
Agreement
Neither agree nor disagree
Mostly disagree
Fully disagree

Fig B: Agreement levels in the cohort of the 22 people who completed both rounds

			Agreement					Change in agreement	
			Round 1		Round 2		Revised		
**Section in R2**	**Characteristic in R1** if changed in R2	**Characteristic in R2**	Min	Opt	Min	Opt	**text?**	Min	Opt
Scope		1. Intended use	95%	81%	95%	95%	No	0%	+ 14%
		2. Target use setting	100%	100%	100%	100%	No	0%	0%
		3. Target users of the app	100%	100%	100%	100%	No	0%	0%
		4. Target population	90%	90%	95%	95%	No	+ 5%	+ 5%
		5. Training requirements	71%	67%	95%	90%	Yes	+ 24%	+ 24%
		6. Ease of use	86%	86%	100%	100%	Yes	+ 14%	+ 14%
System components	8. Compatible mobile devices	7. Compatible mobile devices	86%	81%	90%	95%	Yes	+ 5%	+ 14%
	9. Compatible RDT types	8. Compatible RDT types	95%	81%	90%	95%	Yes	– 5%	+ 14%
	7. Required system components …	9. Additional physical components required	81%	76%	81%	86%	Yes	0%	+ 10%
Functional	(not present)	10. Language support			86%	95%	New		
	10. RDT instructions given by the app	11. Help provided … on how to use the RDT	48%	76%	81%	95%	Yes	+ 33%	+ 19%
		12. Quality control	90%	81%	90%	90%	Yes	0%	+ 10%
		13. Result determination	81%	81%	86%	86%	Yes	+ 5%	+ 5%
		14. Diagnostic data reported	90%	86%	85%	100%	Yes	– 5%	+ 14%
		15. Patient/case data reported	90%	71%	100%	95%	Yes	+ 10%	+ 24%
		16. Contextual data reported	100%	95%	100%	100%	Yes	0%	+ 5%
		17. Methods for user data entry	95%	76%	100%	85%	Yes	+ 5%	+ 9%
		18. User access rights	90%	90%	100%	100%	No	+ 10%	+ 10%
Operational		19. Lighting of the operating environment	86%	81%	95%	85%	Yes	+ 9%	+ 4%
Data		20. Data ownership	71%	67%	90%	95%	Yes	+ 19%	+ 28%
		21. Data flow	81%	81%	100%	100%	No	+ 19%	+ 19%
	21. System access (public API)	22. Data exchange standards	86%	67%	90%	90%	Yes	+ 4%	+ 23%
		23. Handling of intermittent connections	100%	100%	100%	100%	No	0%	0%
		24. Security and privacy	90%	90%	80%	80%	Yes	– 10%	– 10%
		25. Data storage	81%	48%	100%	100%	Yes	+ 19%	+ 52%
Performance		26. Accuracy of results …	90%	86%	90%	90%	Yes	– 0%	+ 4%
Pricing		27. Pricing within the public sector in LMICs	86%	86%	95%	100%	No	+ 9%	+ 14%
		Average	87%	82%	93%	94%	All	+ 6%	+ 13%
							Yes	+ 7%	+ 15%
							No	+ 5%	+ 8%

Level	Count not met		Count not met	
> 50%	1	1	0	0
> 75%	3	5	0	0

Level	Count	
–	4	1
0	7	3
+	15	22

Table D: Additional participant comments relevant to product development

Characteristic (final version)	Summary of comments from all stages
2. Target use setting	• Useful above Level 1 also
7. Compatible mobile devices (smartphones and tablets)	• iOS is rare in these settings
8. Compatible RDT types	• Enable health programs to train the app on their RDTs
10. Language support	• The intended users may not speak the countries' official languages, so ease of adding languages is important
12. Help provided by the app to the user on how to use the RDT	• If allowing the user to set the countdown timer, enforce a minimum time setting so that people do not skip incubation<br>• Be sensitive to the importance of each RDT's regulated labeling<br>• Enable health programs to deploy their own instructions
13. Quality control	• Check for all types of user errors as possible
15. Diagnostic data reported	• Be careful not to over-burden users with data entry<br>• Some health programs will want to queue most photos till the user is on Wi-Fi or another low-cost connectivity method<br>• Ensure users can update or correct results after reporting them
18. Methods for user data entry	• OCR would be nice but is not reliable
19. User access rights	• Depending on design, this may not be necessary
20. Lighting of the operating environment	• "Infrequently", as used in the characteristic description, is vague but no better alternative was found
21. Data ownership	• Many countries lack regulations or policies<br>• Like many items, this needs to be configurable to suit each setting
22. Data flow	• Many countries lack regulations or policies<br>• This could be outside the scope of the app
24. Handling of intermittent connections	• Ask the user before syncing or give the user or admin more control to prevent problems with high use of bandwidth
26. Data storage	• Some health programs will not want to host data in-country because of a lack of infrastructure
