# Supplementary material for: Target Product Profile for a mobile app to read rapid diagnostic tests to strengthen infectious disease surveillance
Source: PLoS One. 2020 Jan 29;15(1):e0228311. doi: 10.1371/journal.pone.0228311 (PMC6988927; doi:10.1371/journal.pone.0228311)
Supplement: S3 Document — As used in feedback round 1. (PDF) [file pone.0228311.s003.pdf]

Supporting Information 3: Draft TPP round 1

As used in feedback round 1

Characteristic	Minimal	Optimal	Notes
**Scope of the app**			
1. Intended use	The app, after photographing each RDT, will suggest an interpretation of the test to the user, who can either accept that or choose an alternate interpretation. The app will transmit these test data as well as patient data entered by the user and contextual data such as the phone's location to the health program.		
2. Target use setting	Community outreach (Level 0) and primary care (Level 1)[1]		
3. Target users of the app	Community health workers with minimal training and any health worker with a similar or superior training level		
4. Target population	Total population presenting for care at relevant settings		
5. Training requirements	Less than ½ hour for existing users of RDTs and mobile devices		
6. Ease of use	< 30 seconds of additional user time per test	No additional user time	Compared to a workflow using paper forms for record-keeping
7. Required system components for the app to be used	• A mobile device that is already available to the user or the organization<br>• An RDT		This app will be compatible with existing RDTs rather than requiring special versions or packages of RDTs
8. Compatible mobile devices	Android mobile devices (smartphones and tablets) that include a camera	Same plus iOS	
9. Compatible RDT types	Qualitative lateral flow tests	Same plus<br><br>Semi-quantitative (threshold) lateral flow tests	These semi-quantitative tests involve comparison of the intensity of a test line to a reference

[1] Ghani AC, Burgess DH, Reynolds A, Rousseau C. Expanding the role of diagnostic and prognostic tools for infectious diseases in resource-poor settings. Nature 2015;528:S50-52

Characteristic	Minimal	Optimal	Notes
**Functional requirements**			
10. RDT instructions given by the app	None	Equivalent to Quick Reference Instructions or a job aid	
11. Quality control	• Check of the RDT's internal control(s)<br>• Check of sufficient capability of the mobile device's camera	Same plus<br><br>• Analysis of background to check sufficient washing of the sample by the buffer<br>• Check of elapsed time to reading of result<br>• Check of expiration by date	Failures of these checks will result in warnings to the user and in the record
12. Result determination	The app suggests the result and requires the user to confirm or override		
13. Diagnostic data reported	• Brand and type of test (possibly by photographing the test and its packaging)<br>• Outcome of quality controls<br>• Result as selected by user<br>• Result as calculated by the app<br>• Photograph used to calculate result, without patient-identifiable information	Same plus<br><br>• Lot number and expiration date of the test (possibly by photographing the test and its packaging)<br>• RDT instruction version<br>• Other relevant diagnostic data as inputted by the user	By removing patient-identifiable information, which might be written on the RDT, from the photograph of the RDT, the photograph can more easily be managed and used for non-clinical purposes like quality control

<table>
<tr><th>Characteristic</th><th>Minimal</th><th>Optimal</th><th>Notes</th></tr>
<tr><td>14. Patient/case data reported</td><td>• Patient identifier: name, ID number, and/or contact info</td><td>Same plus

• Other case information such as symptoms and vital signs
• Additional user information such as age and address</td><td></td></tr>
<tr><td>15. Contextual data reported</td><td colspan="2">• User identification
• Location of test (if enabled by the health program)
• Time and date of test
• Model of mobile device</td><td></td></tr>
<tr><td>16. Methods for user data entry</td><td>• Typing
• Scanning barcodes</td><td>Same plus

• Photographing printed text (optical character recognition (OCR))</td><td>The user can choose from these methods when entering the data types listed above</td></tr>
<tr><td>17. User access rights</td><td colspan="2">Provides access to specific data and app features for users with different roles</td><td>Roles may include data manager (facility supervisor) or RDT user (health care worker)</td></tr>
<tr><td colspan="4">Operational requirements</td></tr>
<tr><td>18. Lighting of the operating environment</td><td colspan="2">Any setting in which the user can see well enough to run the test.

Infrequently, the app may tell the user that it cannot operate in the current lighting, requiring the user to move to easier lighting.</td><td>Example settings:
• dim indoors without artificial lighting
• window-less indoors with fluorescent lighting
• mixed lighting
• outdoors in direct sun
• outdoors in dappled, moving shadows from a tree
• outdoors in shade with indirect sunlight off a red wall</td></tr>
</table>

Characteristic	Minimal	Optimal	Notes
**Data characteristics**			
19. Data ownership	All data collected via the app in the implementation setting is owned by the app owner (i.e. the healthcare program or research establishment)		
20. Data flow	De-identified output data can be exchanged with different authorities with authorization by local authorities		
21. System access (public API)	Publicly available application programming interface (API) for data access protected by authentication and authorization. Technical standards are adhered to.	Same plus<br><br>HIE/HL7 standards are adhered to	For connections to systems such as LISs, DHIS2, EHRs, national registries and surveillance systems
22. Handling of intermittent connections	The user shall be able to perform tests offline, in which case the app shall transmit that data when back online	Same plus<br><br>The app shall synchronize automatically (without user action) in the background when back online	
23. Security and privacy	The app operates under secure connectivity, which meets data protection regulations of individual countries to avoid loss and corruption of sensitive data, and mitigate cyber-attacks, whether data are at rest or in transmission.<br><br>Conforms to national privacy laws.		Encourages GDPR (should no national data security policies exist) to ensure a system that:<br>• preserves data integrity<br>• identifies and mitigates risks<br>• provides relevant parties clear security processes<br><br>Implementation is expected to include processes such as:<br>• Two-factor authentication<br>• De-identified data<br>• Data encryption
24. Data storage	Any data stored in a server/cloud shall be accessible to high-level country authority on a web interface	Any data stored in a server/cloud shall be stored only within that health program's country	

Characteristic	Minimal	Optimal	Notes
**Performance requirements**			
25. Accuracy of results as calculated by the app (for non-clinical purposes)	> 95% concordance with a trained target user	> 98% concordance with a trained target user	
**Pricing and accessibility**			
26. Pricing within the public sector in LMICs	The pricing structure should be adapted to LMICs (including open-source and open-access solutions), and strategies for further cost reductions should be in place		Details to be established in a Global Access agreement. For applicable markets, see https://www.finddx.org/find-negotiated-product-pricing/
